# Notes on Silver Nitrate

**Published:** 1900-04

**Authors:** J. Morgan Howe

**Affiliations:** New York


### NOTES ON SILVER NITRATE.[1]

BY DR. J. MORGAN HOWE, NEW YORK.

EIGHT years of experience in treating teeth with silver nitrate justifies me in taking a few minutes of this meeting to state with some detail that I think the application of this salt to decaying surfaces is one of the most valuable means at our command for treating that condition.

The late Dr. Stebbins rendered the world a great service when he pursued systematic investigations into the effect of treating teeth with this substance, and gave us the results of his experiments, but the benefits to be thus obtained have not yet been appreciated by many.

I have found special advantages in practice from its use, and will briefly refer to desirable results obtainable by such treatment of certain cases of decay.

In the teeth of children, including not only the deciduous teeth but the permanent molars, decay may be arrested without subjecting the child to pain or distress, and this is often a desideratum to the parent and dentist as well as to the patient. The salt is also valuable in the case of the teeth of all whose conditions or necessities forbid their devoting time or energy to the process of having cavities filled.

I have treated the teeth of invalids in this way, with the result of inhibiting decay for several years, and in some cases have filled the largest cavities after the restoration of health, and have been able to note that no progress had occurred in the decay during the period of ill health.

Patients who must leave home, lacking time to have newly formed decay arrested by filling, may often have such inhibition

---

[1] Read before the New York Institute of Stomatology, January 2, 1900.

effected by the same means without detriment. And, furthermore, certain buccal, labial, and lingual cavities have been very satisfactorily treated, in the way under consideration, in the mouths of patients irrespective of conditions of age, health, or purse.

In giving the preference to chemical over mechanical treatment, I have had regard to the conditions of decay on the teeth, and to the liability of such chemically discolored places being exposed to view. Decay affecting the buccal surfaces of the molars of young people is often very superficial, but covers considerable surface, has ill-defined borders, and presents unsatisfactory indications for shaping a cavity which would be at all likely to retain a filling and prevent recurrence of decay.

The advantage of chemically arresting decay in such cases is the certainty with which the silver salt acts upon all the disintegrated tissue, whether it is enamel, dentine, or cementum, converting it into a new compound which acts as a protector to the sound tissues under it. In view of the difficulty of deciding just how far to cut away sound tissue in order to include all the decayed, and the large relative amount of normal tissue that must often be sacrificed to form a cavity for filling, I have often found it an undoubted advantage to treat such cases in the manner under consideration.

Perhaps most of the members of our Institute have been practising similarly, and regard these as elementary suggestions, but I claim the excuse that through our proceedings attention may be called anew to this simple means of arresting decay, which is in danger of being overlooked by the rising generation of dentists.

Avoidance of painful, tedious, and expensive operations, with positive arrest of decay for a time, and conservation of a larger amount of normal tissue, are the advantages of this method. They are offset by certain objections, of which are: unsightly discoloration, liability of pulp irritation in deep cavities, irregularity and roughness of treated surfaces; but the advantages greatly preponderate. Next to preventing the inception of dental decay, we must regard a simple and easily applied method of arresting its progress as a means of serving the many classes of people so circumstanced that the preparation and filling of cavities is undesirable or impossible. Such treatment would have special advantages for soldiers, sailors, travellers, invalids, and all persons whose circumstances preclude payment for proper mechanical treatment. Then, too, there are local conditions, such as I have described, when I think it is only fair that those who are able to pay fees should be allowed the advantages of the treatment indicated.

I have strong hope that this salt of silver will not long remain the only agent possessing the property of acting on decaying tooth-structure so as to form a new stable compound with its organic substance, and so inhibit the destructive process.

I believe that similar properties will be discovered in other substances, without the objectionable discoloring effects, but meantime the use of silver nitrate might be greatly extended with credit to our specialty and with great benefit to humanity.

